# Factors influencing risk perception and nosocomial infection prevention practices of frontline nurses during the COVID-19 pandemic

**DOI:** 10.1186/s12912-021-00591-6

**Published:** 2021-05-17

**Authors:** Xiaoguang Lyu, Jiming Hu, Xin Xu, Yunyan Xianyu, Weiguo Dong

**Affiliations:** 1 The Department of Gastroenterology, Renmin Hospital of Wuhan University, Wuhan, China; 2 School of Information Management, Wuhan University, Wuhan, China; 3 The General Medicine Ward, Renmin Hospital of Wuhan University, Wuhan, China; 4 The Nursing Department, Renmin Hospital of Wuhan University, Wuhan, China

**Keywords:** COVID-19, Nosocomial infection, Risk perception, Preventive practices, Structural equation model

## Abstract

**Background:**

During the coronavirus disease 2019 (COVID-19) pandemic, exploring factors influencing nosocomial infection among frontline nurses may provide evidence to optimize prevention strategies in hospitals.

**Method:**

A large-scale online questionnaire survey of nurses’ state-trait anxiety, job burnout, risk perception, workplace safety perception, knowledge about nosocomial infection, and preventive practices was conducted with 2795 frontline nurses working in the COVID-19 wards of six hospitals in Hubei Province, China, from February 1 to April 1, 2020. The questionnaire data were analyzed using the structural equation modeling (SEM) method to reveal the mechanisms influencing nurses’ risk perception and preventive practices related to nosocomial COVID-19 infection.

**Results:**

A model of the factors that influence nurses’ risk perception and preventive practices regarding nosocomial COVID-19 infection was established. The model verified hypotheses regarding the impact of nurses’ risk perception and preventive practices. Notably, the hypothesis that risk perception has an impact on nurses’ preventive practices regarding nosocomial infection is not valid. Moreover, different marital and educational conditions are associated with significant differences in the impact of state anxiety on the execution of preventive practices, the impact of workplace safety perceptions on risk perception, and the impact of workplace safety perceptions on the execution of preventive practices. The effect of state anxiety on preventive practices differed significantly with different durations of work experience.

**Conclusions:**

According to the results of the influencing factor model, promoting the quality of training on nosocomial infection, meliorating workplace safety, and conducting timely and effective psychological interventions would aid in improving nurses’ preventive practices. Meliorating workplace safety and easing state anxiety would be beneficial to reduce nurses’ risk perception. These strategies are conducive to the optimization of policies for preventing nosocomial COVID-19 infections and similar infectious diseases.

## Background

Coronavirus disease 2019 (COVID-19) is a novel acute infectious respiratory disease caused by severe acute respiratory syndrome coronavirus 2 (SARS-CoV-2) [1]. According to existing clinical studies, COVID-19 is both highly contagious and highly pathogenic, resulting in high intensive care unit (ICU) hospitalization rates and mortality [2]. Currently, the management strategy for patients with mild and moderate COVID-19 is mainly home treatment supplemented with necessary community health services [1], while the strategy for patients with severe COVID-19 includes nosocomial infection prevention, circulation management, respiratory support, multiorgan function assessment, nutrition assessment and support, etc. [2]. Patients with COVID-19 generally have a poor prognosis and a variable reported mortality rate (0–14.6%), and age, underlying diseases, accompanying abdominal pain, breathing difficulty, lymphopenia, and increased D-dimer levels may be risk factors [3].

To date, clinicians have tried various drugs and treatments, mainly from the perspectives of administering antiviral therapy, relieving symptoms, improving respiratory function, reducing inflammation, etc. At present, anti-coronavirus drugs with definite effectiveness are lacking. The existing clinically applied anti-coronavirus drugs mainly include those used against Middle East respiratory syndrome (MERS), severe acute respiratory syndrome (SARS), Ebola virus and influenza virus, such as favipiravir, ribavirin, lopinavir/ritonavir, remdesivir, and arbidol, which are still in clinical trials. The clinical efficacy of these drugs for patients with severe COVID-19 needs to be clinically verified, and their adverse effects must be closely monitored [4, 5]. Clinical experts have also conducted numerous clinical trials on symptomatic treatment. The use of dexamethasone resulted in lower 28-day mortality among those patients who were receiving either invasive mechanical ventilation or oxygen alone but not among those receiving no respiratory support [6]. Hydroxychloroquine/chloroquine did not result in a significantly higher probability of negative conversion but increased the risk of adverse events [7, 8]. Convalescent plasma therapy was also used for critically ill patients; however, its efficacy needs to be verified through further clinical trials [9]. In addition, since the release of the sequence of SARS-CoV-2, scientists have used different technology platforms to develop vaccines, including nucleic acids (DNA/RNA), virus-like particles, peptides, virus vectors (replicable/nonreplicable), recombinant proteins, live attenuated viruses and inactivated viruses [10]. To date, the safety and effectiveness of several new coronavirus vaccines have been verified by large-scale clinical trials, such as the BNT162b2 mRNA COVID-19 vaccine [11], mRNA-1273 SARS-CoV-2 vaccine [12], AZD1222 vaccine [13], and CoronaVac vaccine [14]. Governments have initiated large-scale immunization programs, which is an important step towards the control and termination of the epidemic.

COVID-19 is a global pandemic, and its prevention and control have presented an enormous challenge for clinicians, virologists, public health experts, and government managers [15]. Notably, nosocomial infection with COVID-19 is also a very prominent problem. In January 2020, a study of the clinical characteristics of 138 hospitalized patients with COVID-2019 at a hospital in Wuhan suggested that 41% of the patients developed the disease through nosocomial infection [16]. According to the Chinese Center for Disease Control (CDC), 3019 medical staff members in China contracted COVID-19 during the peak period, 1716 of whom had confirmed cases, while some may have been the result of community infection [17]. Nosocomial infections with COVID-19, especially among medical staff, will seriously affect the ability of hospitals to fight the disease during the epidemic. For hospital managers, further optimization of measures for preventing nosocomial COVID-19 infection is an urgent need.

After surveying the current literature related to COVID-19 prevention, researchers mainly focus on transmission routes, susceptibility factors, and public and nosocomial infection prevention management, among others.

First, to prevent nosocomial infection, understanding the susceptibility factors and transmission pattern of COVID-19, which is a novel acute respiratory infectious disease, is of great significance. In the respiratory care of critically ill patients, factors such as noninvasive ventilation, high-flow nasal intubation, suction, patient transport, etc. increase infection susceptibility [18, 19]. COVID-19 can be spread among family members by asymptomatic patients during social gatherings [20], indicating that COVID-19 nosocomial infections may be partially caused by asymptomatic cases in the hospital [21, 22]. In addition, anesthesia and intensive care services represent risk factors for COVID-19 nosocomial infection among medical personnel [23]. The US CDC has suggested that SARS-CoV-2 is spread by respiratory droplets [24], although the transmission routes of COVID-19 are still disputed among experts. Based on experience in the prevention of COVID-19 nosocomial infection, Wong et al. [25] argued that COVID-19 is not spread through the air in contrast to the understanding of most medical experts; instead, they believe that nosocomial infections among medical staff can be prevented through vigilance and basic infection control measures.

Second, previous studies on COVID-19 infection prevention strategies have mainly focused on public health management and nosocomial infection control. With respect to the prevention and control of COVID-19 in cities through public health management, public health experts have performed fruitful explorations. Some investigators have compared the epidemic patterns of COVID-19 in Guangzhou and Wenzhou and have confirmed that some strict preventive measures, e.g., avoiding large-scale social gatherings, wearing masks, temperature monitoring, restricting the scope of activities, and strengthening quarantine for those with close contact with infected individuals, can effectively control the spread of COVID-19 [26]. With respect to preventing COVID-19 nosocomial infection, many investigators have acquired valuable experience through clinical studies. Hand hygiene has been proven to be the most effective method for preventing COVID-19 nosocomial infection and is inexpensive [27]. Studies by medical personnel working with hemodialysis and burn patients indicated that some practices, e.g., high-quality personal protective equipment (PPE) training, choosing and using PPE correctly when tending to COVID-19 patients, and monitoring the temperature and health of medical staff who are in contact with COVID-19 patients, are effective for preventing nosocomial infection [23, 28, 29]. Moreover, isolation, especially early isolation of COVID-19 patients, was found to effectively prevent nosocomial infection [30] while allowing hospitalized patients to remain in the buffering ward for medical observation (with enhanced traffic control within the hospital) and has become an unconventional means to effectively prevent COVID-19 nosocomial infections [31].

Finally, large-scale surveys on knowledge of COVID-19 prevention, risk perception, and active preventive practices have been conducted to provide evidence for improving preventive measures. According to an online survey of residents in Anhui Province, China, the timely issuance of information about the pandemic by all levels of government and adequate epidemic prevention education enabled residents to have a comprehensive understanding of COVID-19 prevention and adopt good active prevention practices [32]. A survey of medical students in Iran indicated that their grasp of COVID-19-related knowledge significantly affected the students’ active prevention practices, that workplace safety perceptions (WSPs) affected their risk perception, and that risk perception had a negative correlation with the students’ execution of active preventive practices [33]. The above results show that the COVID-19 knowledge, attitudes, and risk perceptions of the surveyed population can predict their active preventive practices, which is very significant for adjustment and optimization of COVID-19 prevention strategies.

During the COVID-19 epidemic, the causes of nosocomial infection among nursing staff were complex. The major influencing factors are as follows: infection prevention practice, knowledge, risk perceptions, WSPs, and psychological factors (anxiety, job burnout, etc.). The impact of the above variables is not clear, but the solution to this problem will provide new evidence for optimization of strategies concerning nosocomial infection prevention and control. Therefore, given the context of widespread concern regarding global epidemic outbreaks and infection prevention and control issues among scientists worldwide, we conducted a large-scale questionnaire survey among nurses and performed modeling to identify the key factors and the mechanism influencing nurses’ risk perceptions and their implementation of preventive practices while focusing on the following questions:

1. Do nurses’ anxiety, job burnout, workplace safety perceptions, and levels of nosocomial infection knowledge significantly affect their risk perception? If yes, to what extent?
2. Do nurses’ nosocomial infection knowledge levels, workplace safety perceptions, anxiety, and risk perceptions significantly affect their preventive practices? If yes, to what extent?

## Methods

### Research model

**Fig. 1:**
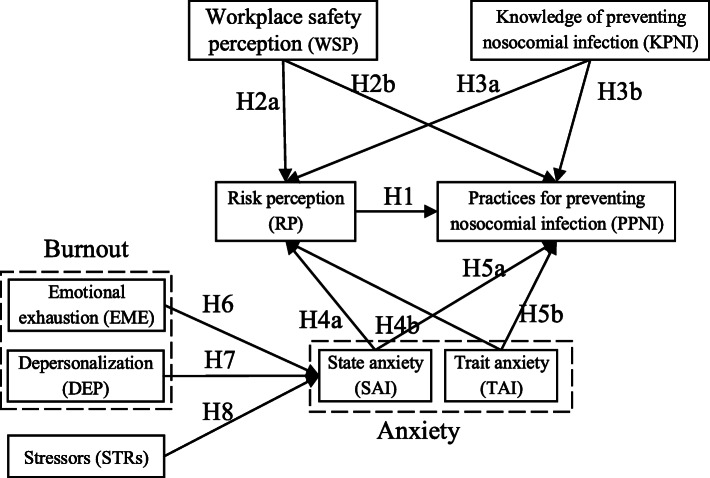
Model of the factors influencing nurses’ risk perception and preventive practices during the COVID-19 pandemic

As described above, in this study, by constructing a nurse-oriented model of factors influencing nurses’ risk perceptions and preventive practices and by processing the survey data, we identified the mechanism influencing nurses' risk perceptions and preventive practices during COVID-19 treatment. Furthermore, based on risk compensation theory, protection motivation theory, and broken window theory in combination with job burnout and psychological anxiety theory, we constructed a model of the factors influencing nurses’ risk perceptions and preventive practices (Fig. 1).

Seven influencing factors or lead variables of risk perception and preventive practice are included in the proposed model: workplace safety perception (WSP); knowledge of preventing nosocomial infection (KPNI); psychological anxiety, including state anxiety (SAI) and trait anxiety (TAI); job burnout, including emotional exhaustion (EME) and depersonalization (DEP); and stressors (STRs).

Meanwhile, the proposed model focused on whether nurses’ risk perception directly affects their preventive practices; whether nurses’ workplace safety perceptions, knowledge of preventing nosocomial infection, state anxiety, and trait anxiety significantly affect their risk perception and corresponding preventive practices; whether nurses’ emotional exhaustion, depersonalization, and stressors significantly affect their state anxiety; and the extent of the potential impact of these factors and which factors are the main factors.

### Model path hypotheses

#### Risk perception

Risk perception refers to an individual’s objective perception of potential harm or loss, i.e., his or her judgment of the attributes and severity of a particular risk [34], which is an important reference for accepting and coping with risk and is affected by various factors, such as personality, knowledge, education, and emotion. According to risk compensation theory and protective motivation theory, risk perception directly or indirectly affects an individual’s behavior. When an individual perceives a high risk, he or she will constantly adjust his or her cognitive processes and choose more protective behavior to avert the risk. In the traffic safety education and public health fields, risk perception has been extensively studied, and risk perceptions have been found to lead to changes in protective measures [35–37].

After preliminary investigations, we found that nurses in China fully appreciated the danger of COVID-19, a novel infectious disease similar to the highly infectious and pathogenic SARS, and have acquired various degrees of risk perception. In addition, the nurses generally adopted necessary COVID-19 prevention practices, such as practicing hand hygiene, correctly selecting and using PPE, avoiding crowds, and implementing quarantine management for COVID-19 patients. According to risk compensation theory and protective theory, the nurses’ level of risk perception may have affected their nosocomial infection prevention practices and may therefore indirectly affect the rate of nosocomial infection with COVID-19.

Based on the above discussion, we propose the following hypothesis:

- **H1: When tending to patients with COVID-19, nurses’ risk perception significantly affects their infection prevention practices**.

#### Workplace safety perception

The broken window effect is a criminological theory, which holds that if a bad behavior in an environment is overlooked, people may feel encouraged to imitate the behavior or even an aggravated version of the behavior. Broken window theory has been fully applied in crime prevention, environmental protection, and education, among others. For example, environmental design has been applied to urban crime prevention in the UK [38]. Tourists’ perceived environment quality will affect their intention to engage in environmentally responsible behaviors [39]. Educators also discovered that if a school is in disorder and has physical risks, its basic educational goals and processes may be jeopardized [40].

The workplace safety perceptions of frontline nurses during the COVID-19 pandemic mainly included reasonable ward zoning, PPE supply, and quarantine management of COVID-19 patients. First, based on previous SARS prevention experience, a modified form of traffic control bundling was adopted to reduce nurses’ exposure to the disease, which is conducive to reducing the probability of nosocomial infection [41]. Second, during the epidemic, PPE was provided to nurses in the ward. PPE, an important aspect of workplace safety, includes masks, protective clothing, face screens, hats, etc. PPE has been confirmed to effectively reduce the nosocomial infection rate by not only isolating the virus and protecting the PPE user but also decreasing the viral load and preventing secondary transmission [42]. Surgical masks played an important role in preventing SARS infection [43]; N95 masks are more effective than surgical masks in reducing the spread of droplets. Medical protective clothing has a strong protective effect on blocking liquid, microorganisms, and aerosols. Third, the management of COVID-19 patients in the ward is an important part of nurses’ workplace safety. When COVID-19 patients walked in the ward, the airflow was found to be significantly contaminated [44], and COVID-19 is spread through droplets and aerosols. Therefore, practicing good quarantine management of COVID-19 patients and limiting their scope of activities reduce the risk of nosocomial spread of the disease.

According to our survey, nurses working on the frontline clearly perceived the basic workplace conditions mentioned above, which affected their risk perception at work. In addition, according to broken window theory, workplace safety perceptions also affect nurses’ work performance and practices for preventing nosocomial infection.

Based on the above discussion, we propose the following hypotheses:

- **H2a: Workplace safety perception significantly affects nurses’ risk perception**.
- **H2b: Workplace safety perception significantly affects nurses’ nosocomial infection prevention practices**.

#### Knowledge level for preventing nosocomial infection

Studies have shown that employees’ understanding of prevention directly affects their occupational safety practices and ultimately has an impact on occupational injuries [45]. Therefore, training nurses on the prevention of nosocomial infection will affect their nosocomial infection prevention practice and thus their nosocomial infection rate.

During the COVID-19 epidemic, hospitals in China conducted timely and comprehensive training on preventing infection that focused on two aspects: (1) common knowledge, such as hand hygiene, proper PPE selection and use, and occupational exposure management; and (2) prevention education focusing on the pathological and infectious characteristics of COVID-19, such as clinical diagnosis, diagnosis and treatment, transmission routes, and patient isolation management [46]. In the preliminary investigation, we found that nurses have strictly complied with the preventive requirements and have constantly adjusted their preventive practices according to their own circumstances and actual care needs.

Based on the above discussion, we propose the following hypotheses:

- **H3a: Nurses’ knowledge regarding the prevention of nosocomial infection significantly affects their risk perception**.
- **H3b: Nurses’ knowledge regarding the prevention of nosocomial infection significantly affects their preventive practices**.

#### State-trait anxiety

Anxiety refers to psychological characteristics represented by nervousness and worry and accompanying physiological changes, such as increased blood pressure [47]. In the field of cognitive psychology, Spielberger proposed the theory of state-trait anxiety [48], where state anxiety refers to an individual’s nervous and anxious emotions in the face of specific dangers or stress events, and trait anxiety is a specific personality or trait that manifests as constant uneasiness and apprehension. Previous studies have confirmed that both state anxiety and trait anxiety have an impact on learning ability, professional behavior, safety and accidents, and risk perception [49–52]; furthermore, studies have also revealed that anxiety is correlated with risk perception and that individuals with high levels of anxiety may perceive an exaggerated risk [53], which is likely to lead to fear or withdrawal behaviors.

During the COVID-19 epidemic, anxiety was an important psychological characteristic of frontline nurses and likely resulted from the following causes: (1) the epidemic outbreak was a major public health emergency event that had an enormous psychological impact on the public; (2) the work intensity of frontline nurses was generally high; (3) the epidemic lasted a long time, and the nurses worked in high-risk posts for long periods, imposing tremendous and continuous psychological pressure on them. This situation persisted for a long time without proper intervention and therefore caused anxiety for the nurses. The nurses’ anxiety can be divided into two categories: (1) state anxiety triggered by high-risk care work in a hospital during the epidemic; and (2) trait anxiety, which is a stable personality characteristic. In this study, we examined the state-trait anxiety of frontline nurses and its impact on their risk perception and nosocomial infection prevention practices. Therefore, we propose the following hypotheses:

- **H4a: Nurses’ state anxiety significantly affects their risk perception**.
- **H4b: Nurses’ state anxiety significantly affects their preventive practices**.
- **H5a: Nurses’ trait anxiety significantly affects their risk perception**.
- **H5b: Nurses’ trait anxiety significantly affects their preventive practices**.

#### Job burnout

According to the World Health Organization (WHO) definition, job burnout refers to the occupational phenomenon caused by chronic work stress that is poorly alleviated. Job burnout is an important factor affecting employees’ health and their pursuit of medical care; however, this condition does not fall within the category of disease [54]. Job burnout mainly manifests as emotional exhaustion, depersonalization, and a low sense of personal accomplishment. Previous studies have confirmed that job burnout may affect employees’ occupational safety practices and can become a work safety hazard. A study of firefighters suggested that those experiencing job burnout show a decreased rate of reporting safety hazards, decreased PPE usage, and a decreased probability of compliance with safety rules [55]. Job burnout was found to be correlated with anxiety, which may be a new mechanism through which job burnout affects occupational safety and job performance. In an investigation of nurses in neonatal intensive care units, job burnout was found to be significantly correlated with anxiety levels [56]. Through a study on police officers, Maria showed that under the intermediary variable of emotional exhaustion, a high-intensity workload can predict anxiety levels [57]. In addition, depersonalization was found to be closely associated with anxiety disorders and depression [58]. To date, a correlation between the low personal accomplishment variable and anxiety has not been reported. According to the survey, when faced with substantial psychological pressure and a high-intensity workload, some frontline nurses did not receive proper psychological intervention, which led to job burnout phenomena such as depersonalization and emotional exhaustion. According to the theoretical bases described above, the job burnout of nurses may indirectly affect their nosocomial infection prevention practices by affecting their state anxiety. Therefore, we propose the following hypotheses:

- **H6: Nurses’ emotional exhaustion significantly affects their state anxiety**.
- **H7: Nurses’ depersonalization significantly affects their state anxiety.**

#### Stressors

According to modern stress effect theory, a stressor is a stimulus that an individual perceives and that generates a positive or negative stress response in a certain job or in certain internal and external environments; when it exceeds a certain limit, the stressor will cause psychological and physiological reactions in the individual, such as insomnia, fear, anxiety, and depression [59, 60]. During the COVID-19 epidemic, public health policies and the work environment changed profoundly and changed the work, family, and personal lives of medical personnel accordingly, which led to changes in nurses’ mental status and thus affected their perceptions of the workplace during the pandemic and the actions that they took in response.

Changes in the stress of nurses during a pandemic have been previously studied. During the SARS and MERS outbreaks, more than 50% of health care workers felt increased work stress [61], with increased negative emotions and concern about their personal safety and their families [62]. During the COVID-19 outbreak and treatment, similar stressors and their effects were also present among nurses. Therefore, we propose the following hypothesis:

- **H8: Nurses’ stressors significantly affect their state anxiety**.

### Empirical method

#### Survey questionnaire design

In this study, we referenced the items of scales used in previous studies and applied appropriate modifications according to the characteristics of the actual work that the nurses performed during the epidemic, ultimately generating the items needed for the questionnaire used in this study. Additionally, we invited 20 experts in the field to evaluate and conduct a pilot test of the draft questionnaire; based on the experts’ feedback, we repeatedly revised and tested the questionnaire.

The questionnaire used in this study included two parts: demographics and scaled questions. First, the nurses’ basic information, including gender, age, education, marital status, job title, department, workplace, etc., was collected. Second, the actual performance of the nurses during the epidemic regarding the factors related to risk perception and preventive practices was examined. For each variable, questions were scored using a 5-level Likert scale (Strongly disagree = 1; Disagree = 2; No opinion = 3; Agree = 4; Strongly agree = 5) to facilitate calculation using structural equation modeling (SEM). The responses to the questions were not scored as right or wrong but aimed to reflect the respondent’s attitude or tendency regarding an item; therefore, the respondents were instructed to answer according to their true perception during the epidemic. The contents of the scaled questionnaire are listed in Table 1.

**Table 1:** Contents of the questionnaire regarding nurses’ risk perception and preventive practices during the epidemic

Variable	Item	Reference
Emotional exhaustion (EME)	EME 1: I feel emotionally drained from my work.	Riley et al. [63]
	EME 2: Working with patients all day in the hospital is a strain for me.	Riley et al. [63]
	EME 3: I feel fatigued when I wake up in the morning and have to face another day on the job.	Riley et al. [63]
	EME 4: I often feel exhausted.	Tang et al. [64]
Depersonalization (DEP)	DEP 1: I feel that I treat some people as impersonal objects.	Riley et al. [63]
	DEP 2: I have become more callous towards people since I took the job.	Riley et al. [63]
	DEP 3: I do not really care what happens to some patients.	Riley et al. [63]
	DEP 4: I try to avoid communicating with patients’ families.	Tang et al. [64]
Stressors (STRs)	STR 1: I felt fear about the wellbeing of my family members during the COVID-19 epidemic.	Khalid et al. [62]
	STR 2: The COVID-19 epidemic has had a substantial impact on my daily life.	Wang [65]
	STR 3: The COVID-19 epidemic has had a substantial impact on my work load and work stress.	Koh et al. [61]
Workplace safety perceptions (WSPs)	WSP 1: The PPE (including the type, quantity, and quality) provided by the hospital is adequate.	Lu et al. [42]
	WSP 2: I think that the management of enhanced traffic control bundling in the hospital is satisfactory.	Schwartz et al. [41]
	WSP 3: I think that the management of the patients in the hospital during the epidemic is satisfactory.	National Health Commission of the PRC [46]
Knowledge of preventing nosocomial infection (KPNI)	KPNI 1: I have a good grasp of knowledge related to COVID-19 infection and treatment. KPNI 2: I have a good grasp of the correct isolation measures and methods for managing patients with COVID-19. KPNI 3: I have a good grasp of knowledge related to hand hygiene. KPNI 4: I can choose and use PPE very well to prevent infection.	Taghrir et al. [33]
Risk perception (RP)	RP 1: I think that I may contract COVID-19 during the epidemic. RP 2: If I contract COVID-19 during the epidemic, I think that my health and life will be seriously threatened. RP 3: Compared with people of the same age and sex, I feel that I am more likely to develop COVID-19 during the epidemic.	Brug et al. [66]
Practices for preventing nosocomial infection (PPNI)	PPNI 1: I perform hand hygiene well when tending to patients. PPNI 2: When tending to patients, I correctly and effectively choose and use PPE (hats, masks, goggles, face screens, etc.).	Lin et al. [67] Lin et al. [67]
	PPNI 3: I have avoided gatherings of medical staff or patients very well.	National Health Commission of the PRC [46]
State anxiety (SAI)	STAI-S	Spielberger and Sydeman [68]
Trait anxiety (TAI)	STAI-T	Spielberger and Sydeman [68]

#### Data collection

The questionnaire survey was conducted from February 1 to April 1, 2020, over a time span of 2 months at hospitals involved in epidemic work, with those in Wuhan as the epicenter. Through the Wenjuanxing online platform, 2797 copies of the questionnaire were collected; 2546 valid copies of the questionnaire were retained after excluding 251 copies with incomplete answers or highly random answers, resulting in a valid questionnaire rate of 91.03%.

#### Ethical approval and informed consent

The study was approved by the Ethical Community of Wuhan University. The respondents of the online survey consented to participate in the study.

## Results

### Descriptive statistical analysis

The statistics of the respondents are shown in Table 2, including gender, age, marital status, job title, position, department, etc.

**Table 2:** Nurses’ basic information

Descriptive statistics	Category	Frequency	Percentage
Gender	Male	77	3.02%
	Female	2469	96.98%
Age	20–30 years	1447	56.83%
	31–40 years	787	30.91%
	41–50 years	229	8.99%
	51–60 years	83	3.26%
	Over 60 years	0	0.00%
Department	Surgery	579	22.74%
	Internal medicine (excluding respiratory medicine)	499	19.60%
	Operating room	288	11.31%
	Obstetrics and gynecology	142	5.58%
	Pediatrics	131	5.15%
	Emergency	130	5.11%
	Critical care	106	4.16%
	Respiratory medicine	104	4.08%
	Infectious diseases	57	2.24%
	Disinfection supply center	37	1.45%
	Other	473	18.58%
Position	Ordinary nurse	2386	93.72%
	Head nurse	160	6.28%
Job title	Junior	1660	65.20%
	Intermediate	803	31.54%
	Senior	83	3.26%
Working years	0–3 years	651	25.57%
	4–5 years	436	17.12%
	6–10 years	646	25.37%
	11–15 years	398	15.63%
	15 years or more	415	16.30%
Education level	Vocational college or below	483	18.97%
	Undergraduate	2024	79.50%
	Master’s degree	39	1.53%
	Ph.D.	0	0.00%
Marital status	Single, without children	951	37.35%
	Married, without children	203	7.97%
	Married, with children	1392	54.67%

In this survey, the number of female nurses was significantly higher than the number of male nurses. Most of the respondents were aged 20–40 years (87.7%), and most were single or married with children. The vast majority had an undergraduate education, a junior or intermediate title, and more than 5 years of work experience, mainly in surgery, internal medicine (excluding respiratory medicine), and the operating room.

### Validity and reliability tests

The survey data were processed with SmartPLS 2.0 software [69] and analyzed by SEM using the partial least squares (PLS) method.

### Reliability test

The reliability test results are shown in Table 3. The overall Cronbach’s α coefficient of the proposed model was 0.937; the Cronbach’s α coefficients of all factors were higher than 0.7, and three factors (i.e., SAI, TAI, and KPNI) had a Cronbach’s α higher than 0.9. The factor loads of all factors were higher than 0.6 and were highly statistically significant (*P* < 0.001), except for TAI3 (*P < 0*.*05*). The composite reliability (CR) values were all greater than 0.8, which was significantly greater than the threshold value of 0.7, with an average variance extracted (AVE) value of 0.53 to 0.83. In summary, all indicators were above their respective threshold values, indicating that the proposed model and the survey data are reliable.

**Table 3:** Model reliability

Construct	Item	Cronbach’s α	Factor loading	*t*-value	Composite reliability (CR)	Average variance extracted (AVE)
DEP	DEP1 DEP2 DEP3 DEP4	0.8049	0.7669 0.8623 0.8494 0.6651	45.2232 79.8495 68.8313 28.8144	0.8679	0.6239
EME	EME1 EME2 EME3 EME4	0.8938	0.8283 0.8799 0.8964 0.8782	96.7024 141.2409 179.8586 149.3751	0.9263	0.7588
KPNI	KPNI1 KPNI2 KPNI3 KPNI4	0.932	0.8411 0.9287 0.9313 0.9421	76.729 220.8435 241.4505 272.3391	0.9516	0.8312
PPNI	PPNI1 PPNI2 PPNI3 PPNI4	0.8989	0.8527 0.8816 0.9061 0.8627	86.0582 124.0698 154.3556 89.7469	0.9295	0.7674
RP	RP1 RP2 RP3	0.7375	0.6385 0.9216 0.9171	6.9654 145.0625 115.3461	0.8719	0.6994
SAI	SAI1 SAI2 SAI3 SAI4 SAI5 SAI6 SAI7 SAI8 SAI9 SAI10 SAI11 SAI12 SAI13 SAI14 SAI15 SAI16 SAI17 SAI18 SAI19 SAI20	0.9525	0.7104 0.7277 0.7096 0.6972 0.7678 0.7386 0.7305 0.6326 0.7987 0.666 0.6889 0.7188 0.7793 0.6522 0.7612 0.7446 0.7505 0.7812 0.6989 0.7115	50.1333 57.0518 52.8561 53.2656 66.9915 58.4887 54.8664 36.1604 94.4711 45.436 46.8967 55.3918 75.8216 42.6972 55.6441 55.4014 62.0756 74.05 42.0313 47.7655	0.9144	0.5174
STR	STR1 STR2 STR3	0.7551	0.7541 0.8095 0.872	26.3306 37.3919 40.8764	0.8538	0.6615
TAI	TAI1 TAI2 TAI3 TAI4 TAI5 TAI6 TAI7 TAI8 TAI9 TAI10 TAI11 TAI12 TAI13 TAI14 TAI15 TAI16 TAI17 TAI18 TAI19 TAI20	0.9162	0.7587 0.727 0.7662 0.7728 0.6887 0.7843 0.7237 0.6993 0.7103 0.6969 0.7028 0.7309 0.7318 0.7288 0.7184 0.7349 0.7139 0.7716 0.7791 0.775	66.0359 28.5618 2.045 41.2477 37.2915 40.7696 44.072 40.1196 40.0023 6.6303 45.3725 47.3958 22.4037 33.5663 37.4342 40.5243 48.0227 58.4849 39.8821 43.5097	0.9208	0.5381
WSP	WSP1 WSP2 WSP3	0.7615	0.8969 0.723 0.6799	95.2758 21.8092 8.5427	0.8137	0.596

### Validity test

In terms of content validity, the theories and models on which this study was based are extensively used, and the measurement indicators were all derived from previous research results and appropriately modified according to the actual situation; therefore, the content validity of this study is strong. Table 4 shows the correlation coefficients between variables and the square root values of the AVE; except for the high correlation coefficient between SAI and TAI, the correlation coefficients between the other variables were significantly lower than the respective square root values of the AVE, indicating that the validity of the model established in this study is high.

**Table 4:** Correlation coefficients of influencing factors and their AVE square roots

	DEP	EME	KPNI	PPNI	RP	SAI	STR	TAI	WSP
DEP	***0.7899***								
EME	0.4092	***0.8711***							
KPNI	−0.1746	−0.1550	***0.9117***						
PPNI	−0.2278	− 0.2133	0.4811	***0.8760***					
RP	0.0790	0.2681	−0.0531	−0.0877	***0.8363***				
SAI	0.2661	0.4820	− 0.1237	− 0.1813	0.3046	***0.7193***			
STR	0.0752	0.1282	0.0128	−0.0285	0.1240	0.1543	***0.8133***		
TAI	0.3227	0.4685	−0.1634	− 0.2320	0.2633	*0.6493*	0.1508	***0.7336***	
WSP	−0.1726	−0.2978	0.3707	0.4975	−0.2189	−0.2795	− 0.0382	−0.2636	***0.7720***

### Model calculation results

**Fig. 2:**
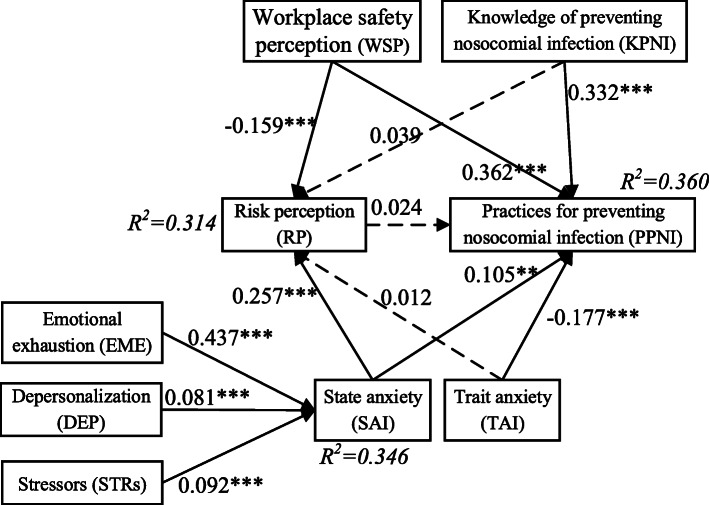
Calculation results of the model of factors influencing nurses’ risk perception and nosocomial infection prevention practice during the epidemic. Notes: The solid line indicates that the path assumption is tenable, while the dashed line indicates that the path assumption is not tenable. The asterisk * represents the degree of significance. *P < 0.05; **P < 0.01; ***P < 0.001

The calculation results indicate that the hypotheses regarding the model of factors influencing nurses’ risk perception and preventive practices, the questionnaire design, and the hypotheses regarding the fit between the two were verified, indicating that the proposed model can solve the research issues in a targeted manner. The calculation results of the model path hypotheses are shown in Fig. 2 and Table 5.

**Table 5:** Calculation results regarding the influencing relationship path hypotheses

Hypotheses	Standardized coefficient	*t*-value	Supported
H1 (RP → PPNI)	0.024	1.3616	*No*
H2a (WSP → RP)	−0.159	6.5439	Yes
H2b (WSP → PPNI)	0.362	16.5995	Yes
H3a (KPNI→RP)	0.039	1.6592	*No*
H3b (KPNI → PPNI)	0.332	12.0851	Yes
H4a (SAI → RP)	0.257	6.3944	Yes
H4b (SAI → PPNI)	0.105	3.2554	Yes
H5a (TAI → RP)	0.012	0.2387	*No*
H5b (TAI → PPNI)	−0.177	5.135	Yes
H6 (EME → SAI)	0.437	23.5433	Yes
H7 (DEP → SAI)	0.081	4.111	Yes
H8 (STR → SAI)	0.092	5.5261	Yes

Although the hypotheses of the Hl (RP → PPNI), H3a (KPNI → RP), and H5a (TAI → RP) paths were invalid, the hypotheses of the other paths were all valid, with a significance level of *P <* 0.001. First, the preventive practices adopted by the nurses were not significantly affected by their risk perceptions, but they were significantly positively affected by their workplace safety perceptions (*β* = 0.362, *P* < 0.001), knowledge of preventing nosocomial infection (*β* = 0.332, *the p* < 0.001), and state anxiety (*β* = 0.105, *P* < 0.05) and significantly negatively affected by trait anxiety (*β* = − 0.177, *P* < 0.001). Second, throughout the course of their work, the nurses’ risk perceptions were significantly positively affected by state anxiety (*β* = 0.257, *P* < 0.001) and significantly negatively affected by workplace safety perceptions (*β* = − 0.159, *P* < 0.001), while the training provided to the nurses by the hospital and the nurses’ trait anxiety did not significantly influence their risk perceptions. Third, when providing nursing care, the nurses’ emotional exhaustion (*β* = 0.437, *P* < 0.001), depersonalization (*β* = 0.081, *P* < 0.001), and stressors (*β* = 0.092, *P* < 0.001) significantly positively affected the resulting state anxiety. Notably, the nurses’ emotional exhaustion was the most prominent influencing factor. The overall epidemic protection and treatment efforts caused them to experience profound emotional depletion, which quickly affected their mental state and led to a high level of state anxiety.

In terms of the model’s overall analytical power, the R^2^ value of the nurses’ nosocomial infection prevention practices was 0.36, indicating that the main variables (risk perception and state anxiety) have a comprehensive explanation capacity of 36%, representing moderate analytical power.

### Moderating role of model assumptions

We analyzed differences in the influence paths of job title, marital status, education, and working years, and the results are shown in Table 6.

**Table 6:** Differential analysis of the influence paths

Path	Grouped data		*t* -value
WSP * title → RP	Junior title	−0.154	5.1227
	Intermediate title	−0.147	
	Senior title	−0.197	
WSP * title → PPNI	Junior title	0.335	2.8277
	Intermediate title	0.396	
	Senior title	0.372	
SAI * marriage → PPNI	Single	−0.003	3.5409
	Married without children	−0.001	
	Married with children	0.164	
WSP * marriage → RP	Single	−0.204	4.6259
	Married without children	−0.193	
	Married with children	−0.125	
WSP * marriage → PPNI	Single	0.341	2.7268
	Married without children	0.289	
	Married with children	0.383	
SAI * edu → RP	Vocational college or below	0.397	2.4221
	Undergraduate	0.245	
	Master’s	0.049	
	Ph.D.	N/A	
WSP* edu → RP	Vocational college or below	−0.106	8.8948
	Undergraduate	− 0.156	
	Master	−0.455	
	Ph.D.	N/A	
WSP * edu → PPNI	Vocational college or below	0.335	3.6723
	Undergraduate	0.361	
	Master	0.597	
	Ph.D.	N/A	
WSP * seniority → PPNI	0–3 years	−0.021	3.109
	4–5 years	−0.065	
	6–10 years	0.086	
	11–15 years	0.289	
	15 years or more	0.221	

In terms of job title, the effect of WSPs on risk perception and the effect of WSPs on PPNI differed. Nurses with a senior title showed the strongest risk perception, while those with an intermediate title adopted the best nosocomial infection prevention practices.

In terms of marital status, the influence paths of SAI → PPNI, WSP → RP, and WSP → PPNI differed. Notably, the effect of state anxiety on the adoption of nosocomial infection prevention practices was low among nurses who were single and married without children but high among those who were married with children. Compared with the married group, workplace safety perceptions had a greater impact on risk perception among the single nurses but a greater impact on the adoption of nosocomial infection prevention practices among those who were married with children.

In terms of education level, the influence paths of SAI → PPNI, WSP → RP, and WSP → PPNI differed. Notably, because the number of nurses with a master’s degree or above was low, conducting a comparative analysis was inappropriate. State anxiety had a greater impact on risk perception among nurses with an education level of vocational college or below, while workplace safety perceptions had a greater impact on risk perception and the adoption of nosocomial infection prevention practices among nurses with an undergraduate education.

The influence path of SAI → PPNI differed according to work experience. State anxiety had a greater impact on the adoption of nosocomial infection prevention practices among nurses with more than 11 years of work experience and had the greatest impact on those with 11–15 years of work experience, while it had a low impact on the adoption of nosocomial infection prevention practices among nurses with fewer than 10 years of work experience.

## Discussion

In this study, we successfully established a model of factors influencing nurses’ risk perception and adoption of nosocomial infection prevention practices. We further examined the variables, including mental health factors, workplace safety perceptions, and knowledge of nosocomial infection, and their influence on nurses’ risk perception and preventive practice. After collecting and analyzing survey data from nurses, we drew some conclusions that have implications for the optimization of hospital management.

### Major findings

#### First, we found that the quality of nosocomial infection prevention management has a significant impact on nurses’ nosocomial infection prevention practices, i.e., workplace safety and training on the prevention of nosocomial infections affect nurses’ nosocomial infection prevention practices

(1) Workplace safety perceptions have the most significant positive impact on nurses’ nosocomial infection prevention practices. Consistent with broken window theory, a good working environment and safety management have a positive impact on nurses’ work quality. Manapragada et al. [70] showed that the safety climate of medical staff is highly positively correlated with their safety performance, which supports our findings. (2) The behavior model constructed in this study suggests that nurses’ grasp of nosocomial infection prevention knowledge has a significantly positive impact on their adoption of nosocomial infection prevention practices. Previous studies have suggested that nurses’ knowledge of nosocomial infection prevention may affect their attitude and thus may ultimately indirectly affect their nosocomial infection prevention practice [71]. Therefore, providing training for COVID-19 frontline nurses regarding nosocomial infection prevention will help them improve their basic prevention skills. Judith showed that when medical personnel have full knowledge of nosocomial infection prevention and hand hygiene supplies are adequately available in the workplace, the hand hygiene compliance of personnel significantly improves [72]. A study on the relationship between the knowledge and practice of preventing infection through quarantine conducted by Suliman et al. [73] showed that although medical staff had good knowledge of isolation management, they exhibited poor implementation compliance. Therefore, we speculate that the nosocomial infection safety practices of nurses are jointly affected by their knowledge of nosocomial infection prevention and workplace safety perceptions, and the single factor of knowledge of nosocomial infection prevention does not directly lead to good safety practices.

#### Second, we found that psychological factors such as state-trait anxiety and job burnout are important factors affecting nurses’ nosocomial infection prevention practice

(1) A moderate level of state anxiety can positively affect nurses’ nosocomial infection prevention practice, i.e., the quality of nosocomial infection prevention practices among nurses with relatively higher state anxiety is better than that among nurses with low state anxiety. Previous studies on workplace anxiety have indicated that anxiety has both positive and negative aspects. Excessive anxiety affects employees’ work performance, but moderate levels of anxiety have a stimulating effect on employees, increasing their receptivity to feedback regarding their work performance and thus their vigilance in terms of the supervision and management that they receive, which leads to better performance [74, 75]. Based on the behavioral model proposed in this study, the nurses’ state anxiety during the epidemic was at an appropriate level and had a positive effect on their nosocomial infection prevention practices. Therefore, heeding employees’ state anxiety levels, implementing effective measures to reduce state anxiety (e.g., promoting successful COVID-19 prevention and treatment, strengthening organizational support and psychological counseling interventions for nurses), and maintaining nurses’ state anxiety levels within a moderate range are issues warranting the attention of hospital managers. (2) Interestingly, trait anxiety negatively predicts nurses’ nosocomial infection prevention practices, i.e., higher trait anxiety levels correspond to lower execution of nosocomial infection prevention practices. According to state-trait anxiety theory, trait anxiety is a longer-lasting and more stable personality trait. Under the same stress scenario, individuals with high trait anxiety are more prone to experiencing more anxious emotions with high stability [48]. Previous studies have suggested that employees with high trait anxiety have poor work performance [75] and that the level of state anxiety can predict employees’ inattention in dangerous situations [76]. These studies suggest that frontline nurses with high levels of trait anxiety should concentrate on their work when working in high-risk areas to reduce work errors and occupational exposure risks. Hospital administrators must direct attention towards employees with high levels of trait anxiety, e.g., by strengthening training on the prevention of nosocomial infections, through psychological intervention, etc., to reduce their risk of nosocomial infection. (3) During the COVID-19 epidemic, nurses’ long-term work stress and work intensity significantly increased, resulting in varying degrees of job burnout. Emotional exhaustion and depersonalization are two important dimensions of job burnout. Emotional exhaustion manifests as an employee’s loss of enthusiasm and work motivation, while depersonalization manifests as indifference and negligence towards work objects and environments. Manomenidis et al. [77] found that job burnout can reduce nurses’ hand hygiene compliance, which also verifies our findings. In the proposed model of factors affecting nurses’ nosocomial infection prevention practices, we found that the depersonalization and emotional exhaustion associated with job burnout can indirectly affect nurses’ nosocomial infection prevention practice by affecting their state anxiety.

#### Third, we found that nurses’ workplace safety perceptions and state anxiety have a significant impact on their risk perception

Risk perception during the COVID-19 epidemic is an important psychological feature of nurses working on the frontline in the fight against the epidemic and an important factor influencing their mental health, work performance, and job change intention. In this study, we focused on factors influencing nurses’ risk perception and found the following two important influence paths: (1) Nurses’ safety perception of the ward environment has a significant influence on their risk perception. Safety climate refers to an employee’s intuitive perception of the degree to which his or her company values safety and determines the employee’s occupational safety practices and initiative in participating in safety management [78, 79]. A survey of US construction workers showed that in a more active workplace safety climate, workers demonstrate a higher hazard identification capability and a higher degree of risk perception [14]. In the proposed SEM, nurses’ assessment of workplace safety includes the appropriateness of ward division, PPE availability, the quality of isolation and management of COVID-19 patients, etc., and the workplace safety variable represents the safety climate of the hospital to a certain extent. Therefore, that the workplace safety perceptions of the frontline nurses would have a significant influence on their risk perception is a reasonable finding. (2) Nurses’ state anxiety significantly affects risk perception. Previous studies have suggested that employees’ mental health is an important factor in risk perception. A survey of the relationship between mental state and risk perception showed that the anxiety and paranoid personality group was more likely than the control group to think that negative events occur with high probability [80]. Therefore, nurses may be overly concerned about adverse events during the COVID-19 epidemic while overestimating the risk of COVID-19, which may be the reason why state anxiety affects nurses’ risk perception.

In addition, in the proposed model, three invalid hypotheses are noteworthy. First, the hypothesis that risk perception affects the execution of nosocomial infection prevention practices is invalid. A study of nurses’ risk perception, knowledge, and prevention practices regarding occupational exposure to the Zika virus indicated that risk perception and knowledge of nosocomial infection prevention can ultimately affect nurses’ preventive practices by influencing their attitude towards prevention [71]. According to our hypothesis, under perceptions of high risk, nurses are expected to implement nosocomial infection prevention measures highly efficiently. The reason for this invalid influence path may be that (1) in the early stage of the epidemic, some nurses did not have a positive attitude regarding the prevention of nosocomial infection, (2) PPE was not readily available, and although the nurses had appropriate risk perceptions at the time, they were unable to adopt the corresponding high-quality nosocomial infection prevention practices because of a lack of appropriate materials. Second, the hypothesis of the influence path between knowledge of nosocomial infection prevention and risk perception was invalid, likely because training on the prevention of nosocomial infection was dominated by knowledge about how to effectively prevent COVID-19 and only minimally addressed the risk, prognosis, and other information; therefore, this factor was unable to influence nurses’ risk perception. Third, the influence path between trait anxiety and risk perceptions of COVID-19 was invalid, although the reasons for this result are unclear. We speculate that employees with trait anxiety may be distracted and thus unable to accurately assess workplace safety, which leads to the invalidity of the hypothesis of this influence path.

#### Fourth, we performed differential analyses at the demographic level

Based on the nurses’ education, job title, working years, and marital status, we performed a differential analysis of the influence paths and found that the effects of trait anxiety and workplace safety perceptions on risk perception and nosocomial infection prevention practices differed.

First, as the nurse’s education level and job title increased, the positive impact of workplace safety perceptions on risk perception and nosocomial infection prevention practices gradually increased for the following reasons: education level and job title reflect nurses’ knowledge level and work ability to a certain extent, and those with high academic qualifications and high professional titles may be better able to observe and make judgements regarding workplace safety based on their better knowledge of nosocomial infection. These more experienced nurses also have better clinical capabilities and a greater capability to execute nosocomial infection prevention practices. Therefore, among nurses with a high professional title and a higher education level, the risk perception and the broken window effect of workplace safety perceptions are more profound.

Second, risk perception and nosocomial infection prevention practices differed significantly between nurses who were single and those who were married with children because the latter group has more complex family relations, causing them to worry about the health of their families and children in addition to their own health. These factors increase the intensity of stressors in nurses with more work experience; additionally, the level of state anxiety increases accordingly but remains within an appropriate range, resulting in increased execution of nosocomial infection prevention practices.

Additionally, we found that with an increase in work years, the positive influence of state anxiety on the execution of nosocomial infection prevention practices increased significantly because nurses with more work experience are affected by other variables, such as marital status, and are most often married or married with children, which has a moderating effect similar to that of marital status.

### Limitations

The model of nurses’ implementation of nosocomial infection prevention practices has some limitations: (1) The anonymity of participants is an important issue to protect the privacy of participants and ensure the validity of the research results. Effective measures should be taken to ensure the anonymity of the questionnaire participants. (2) The nosocomial infection prevention practices variable in the model is based on nurses’ self-reports, which are subjective, and its conformity with the actual situation has not been verified. (3) The model is incomplete since an individual’s risk perception is also affected by various factors, such as information sources and emotional factors (e.g., fear, depression, stress), and because the outcome of risk perception includes fear, withdrawal, and other response indicators that are not included in the model and will be addressed in the future. (4) In this study, we used SmartPLS for our SEM, and this method has various problems, such as low parameter estimation accuracy. Therefore, the results of this study need to be further verified and adjusted in hospital management practice.

## Conclusions

We successfully established a model to identify the important factors influencing the risk perception and nosocomial infection prevention practices of frontline nurses. The results revealed by the model are beneficial for nurses to improve their preventive practices when working with patients with COVID-19. On the other hand, our discoveries are also useful to limit the risk perception of frontline nurses, which is beneficial to nurses’ mental health and qualified employee retention in medical services during the COVID-19 pandemic. We recommend that hospital managers adopt the following policies for preventing nosocomial infections with COVID-19:

1. Improving workplace safety through a series of management measures, such as providing adequate and reliable PPE, rationally zoning the ward, and strictly isolating COVID-19 patients, can encourage nurses to implement nosocomial infection prevention practices.
2. Strengthening training on basic knowledge of COVID-19 and nosocomial infection prevention can improve nurses’ nosocomial infection prevention practices. Management improvement measures can include standardizing hand hygiene practice, properly choosing and using PPE, providing training on the quarantine and management of COVID-19 patients, and assessing nurses’ knowledge regarding nosocomial infection, among others.
3. Heeding nurses’ mental health problems, such as anxiety and job burnout, providing mental health interventions in a timely manner, and offering full care and organizational support will help nurses reduce their risk perception and improve their execution of nosocomial infection prevention practices. Special attention should be directed towards nurses with more than 11 years of work experience regarding job burnout, anxiety, and other mental conditions.

## Data Availability

The data supporting the findings of this study are available from the Information Security Center, Renmin Hospital of Wuhan University, but restrictions apply to the availability of these data, which were used under license for the current study and are therefore not publicly available. However, data are available from the authors upon reasonable request and with permission from the Research Management Department, Renmin Hospital of Wuhan University.

## Abbreviations

SARS: Severe Acute Respiratory Syndrome Coronavirus
MERS: Middle East Respiratory Syndrome Coronavirus
PLS: Partial Least Squares
SEM: Structural Equation Modeling
CR: Composite Reliability
AVE: Average Variance Extracted
CDC: Center for Disease Control and Prevention
PPE: Personal Protective Equipment
WSP: Workplace Safety Perception
KPNI: Knowledge of Preventing Nosocomial Infection
SAI: State Anxiety
TAI: Trait Anxiety
EME: Emotional Exhaustion
DEP: Depersonalization
STR: Stressor
